# Pancreatic adenocarcinoma response to chemotherapy enhanced with non-invasive radio frequency evaluated via an integrated experimental/computational approach

**DOI:** 10.1038/s41598-017-03040-0

**Published:** 2017-06-13

**Authors:** Matthew J. Ware, Louis T. Curtis, Min Wu, Jason C. Ho, Stuart J. Corr, Steven A. Curley, Biana Godin, Hermann B. Frieboes

**Affiliations:** 1 Department of Surgery, Baylor College of Medicine, Houston, TX USA; 2 Department of Bioengineering, University of Louisville, Louisville, KY USA; 3 Department of Engineering Sciences and Applied Mathematics, Northwestern University, Chicago, IL USA; 4 Department of Chemistry and Smalley-Curl Institute, Rice University, Houston, TX USA; 5 Department of Bioengineering, University of Houston, Houston, TX USA; 6 Department of Nanomedicine, Houston Methodist Research Institute, Houston, TX USA; 7 James Graham Brown Cancer Center, University of Louisville, Louisville, KY USA

## Abstract

Although chemotherapy combined with radiofrequency exposure has shown promise in cancer treatment by coupling drug cytotoxicity with thermal ablation or thermally-induced cytotoxicity, limited access of the drug to tumor loci in hypo-vascularized lesions has hampered clinical application. We recently showed that high-intensity short-wave capacitively coupled radiofrequency (RF) electric-fields may reach inaccessible targets* in vivo*. This non-invasive RF combined with gemcitabine (Gem) chemotherapy enhanced drug uptake and effect in pancreatic adenocarcinoma (PDAC), notorious for having poor response and limited therapeutic options, but without inducing thermal injury. We hypothesize that the enhanced cytotoxicity derives from RF-facilitated drug transport in the tumor microenvironment. We propose an integrated experimental/computational approach to evaluate chemotherapeutic response combined with RF-induced phenotypic changes in tissue with impaired transport. Results show that RF facilitates diffusive transport in 3D cell cultures representing hypo-vascularized lesions, enhancing drug uptake and effect. Computational modeling evaluates drug vascular extravasation and diffusive transport as key RF-modulated parameters, with transport being dominant. Assessment of hypothetical schedules following current clinical protocol for Stage-IV PDAC suggests that unresponsive lesions may be growth-restrained when exposed to Gem plus RF. Comparison of these projections to experiments *in vivo* indicates that synergy may result from RF-induced cell phenotypic changes enhancing drug transport and cytotoxicity, thus providing a potential baseline for clinically-focused evaluation.

## Introduction

Pancreatic ductal adenocarcinoma (PDAC) is a highly lethal disease characterized by poor chemotherapeutic response. In particular, the effects of chemotherapy for PDAC are hampered by low vascularization, hypoxia and diffusive transport limitations in the tumor microenvironment^1–4^. Clinical trials have shown that inefficient drug transport in PDAC lesions can be directly correlated to irresponsiveness to therapy^3^. Some trials have combined gemcitabine (Gem) with ionizing radiation and other therapeutic modalities^5, 6^, but have failed to substantially improve the response or overall survival compared to Gem alone.

We have recently reported that combining Gem therapy with a novel, non-invasive technology based on high-intensity short-wave capacitively coupled radiofrequency (RF) electric-fields^7–9^ significantly enhances therapeutic efficacy when compared to either non-invasive RF or Gem. Unlike clinically used RF ablation modalities, which rely on insertion of electrodes into the tumor tissue followed by thermal ablation of both cancerous and healthy tissue at 350–500 KHz, this non-invasive RF is operated using 13.56 MHz frequency, which is one of the frequencies internationally selected for Industrial, Scientific and Medical (ISM) applications, and is considered safe for body tissues. This novel technology uniquely affects PDAC cell phenotype, and, thus, may have the potential to overcome chemotherapeutic drug diffusive transport limitations^10^. Specific effects observed *in vitro* include changes to cell-cell adhesion, elasticity and morphology, which offer the potential to affect drug transport, distribution, and accumulation within tumor tissue^10^. Additionally, we have recently shown that exposure to this RF method increases interstitial transport and perfusion of fluorescent probes across tumor-associated vasculature^11^.

It is unclear, however, how the cell-scale phenotype changes observed with exposure to the non-invasive RF technology relate to the favorable chemotherapeutic tissue-scale response observed *in vivo* ^7–9^. Although numerous studies have indicated the ability of hyperthermia to enhance potency of standard chemotherapy^12, 13^ as well as radiation therapy^14^, our technology has been shown to not rely on hyperthermia *per se* to kill tumor cells, but rather on cellular-scale phenotypic changes that enhance chemotherapeutic efficacy^7, 10, 11, 15–17^. This stands in contrast to traditional techniques, such as radiofrequency ablation (RFA), in which a probe is invasively inserted into the diseased site and high heat (50–100 °C^18, 19^ is administered, causing coagulative necrosis and tissue thermal destruction^20^, or more recent non-invasive developments relying on hyperthermia for the cytotoxic effect^21, 22^.

Here, we evaluate how the cell-scale changes induced by RF may help to overcome chemotherapeutic diffusive transport limitations of Gem^23^ in the hypovascularized microenvironment of PDAC lesions. We hypothesize that phenotypic changes in PDAC cells observed when in optimal drug exposure (monolayer cell culture) as a result of RF therapy^10^ would also affect the response in the 3D microenvironment subject to impaired transport. We employ a 3D cell culture system (tumor spheroids) to isolate and study these effects. As the complex spatial-temporal interactions between tissue and therapy parameters preclude evaluation of treatment response solely through experimental analysis, we further propose computational modeling to simulate the effects of these parameters on therapy outcome, and compare the predicted tumor response to the results observed in an ectopic mouse tumor model *in vivo*.

## Results

### Cell viability decreases after non-invasive RF plus Gem in optimal transport (monolayer) conditions

**Figure 1:**
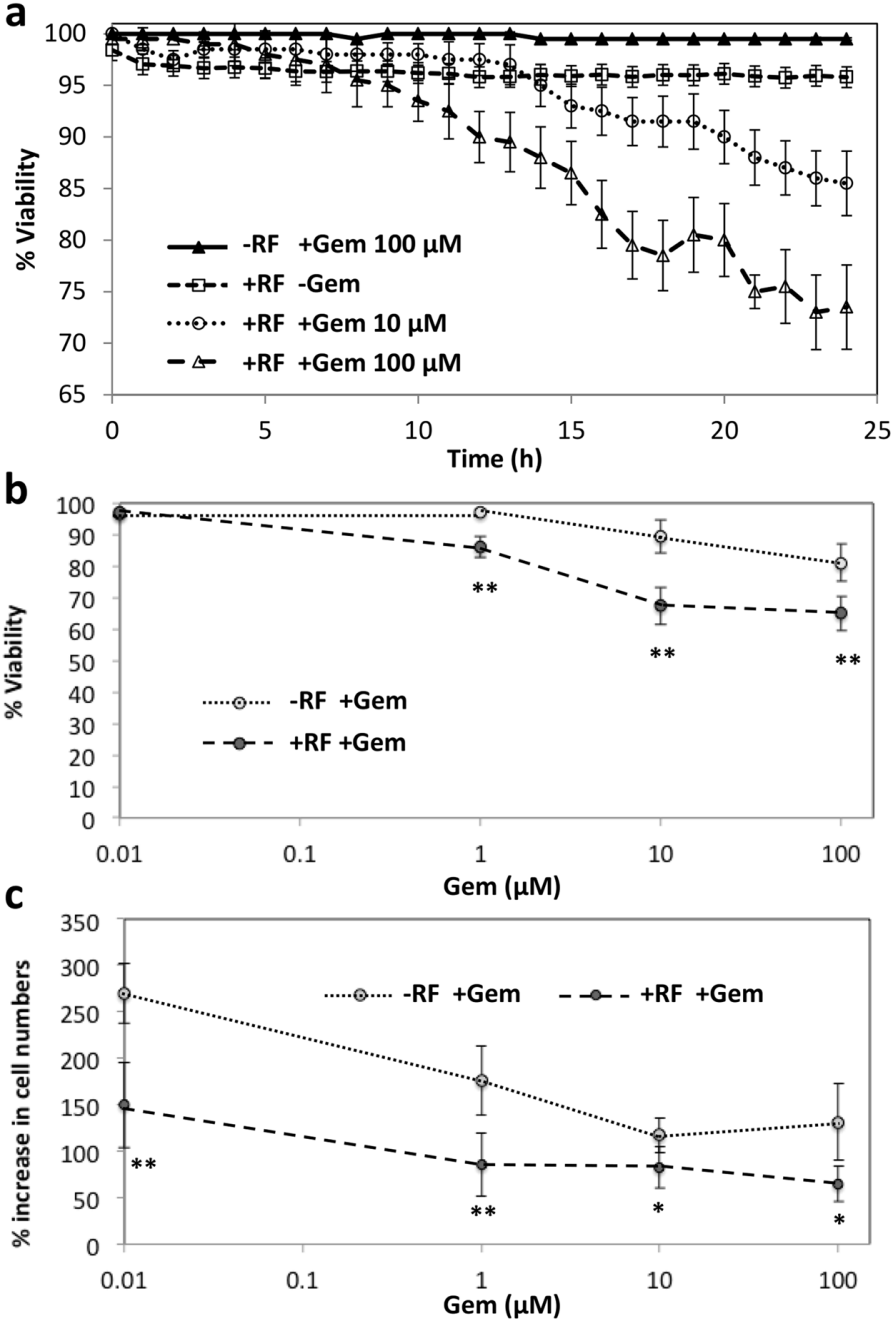
Combination treatment with Gem and non-invasive RF field exposure yields mildly enhanced cytotoxicity and cytostaticity as compared to Gem alone in PDAC monolayer cell culture. (**a**) Percent viability of PANC-1 cells treated with various combinations of RF and Gem; (**b**) Percent viability of PANC-1 cells treated with Gem alone and Gem+RF at 48 h; (**c**) Percent cytostaticity of PANC-1 cells treated with Gem alone and Gem+RF at 48 h. In all cases Gem was administered immediately after RF exposure (mean ± SD, n = 6). Statistical significance determined using two-tailed Student’s t-test with significance level 0.05 (*) or 0.01 (**).

RF exposure immediately before dosing with Gem causes viability decrease (Fig. 1): exposure of PDAC cells to 100 μM Gem or RF plus 100 μM Gem, yields 98% and 73% viability at 24 h, respectively (Fig. 1a). Viability decreases further to 85% in 100 μM Gem dosed cells and 65% in RF plus 100 μM dosed cells at 48 h (Fig. 1b). Cytostatic effect is observed for exposure to RF plus Gem, with increase in cell number of 60% at 48 h compared to 140% increase when dosed without RF (Fig. 1c). We previously determined that after a single non-invasive RF treatment, space between cells (cell-free substrate region) increased by ~24%; however, after 4 treatments it only increased by ~4%^10^. We also found that when cells which detach following RF (present in the supernatant) are reseeded in fresh media, they remain susceptible to thermal shock as nearly 100% of cells in this sub-population are rounded after the exposure^10^. Further, the cell proportion affected by the exposure as indicated by their roundness declined by 10% after four RF treatments^10^, suggesting the development of resistance.

### Non-invasive RF affects molecule transport

**Figure 2:**
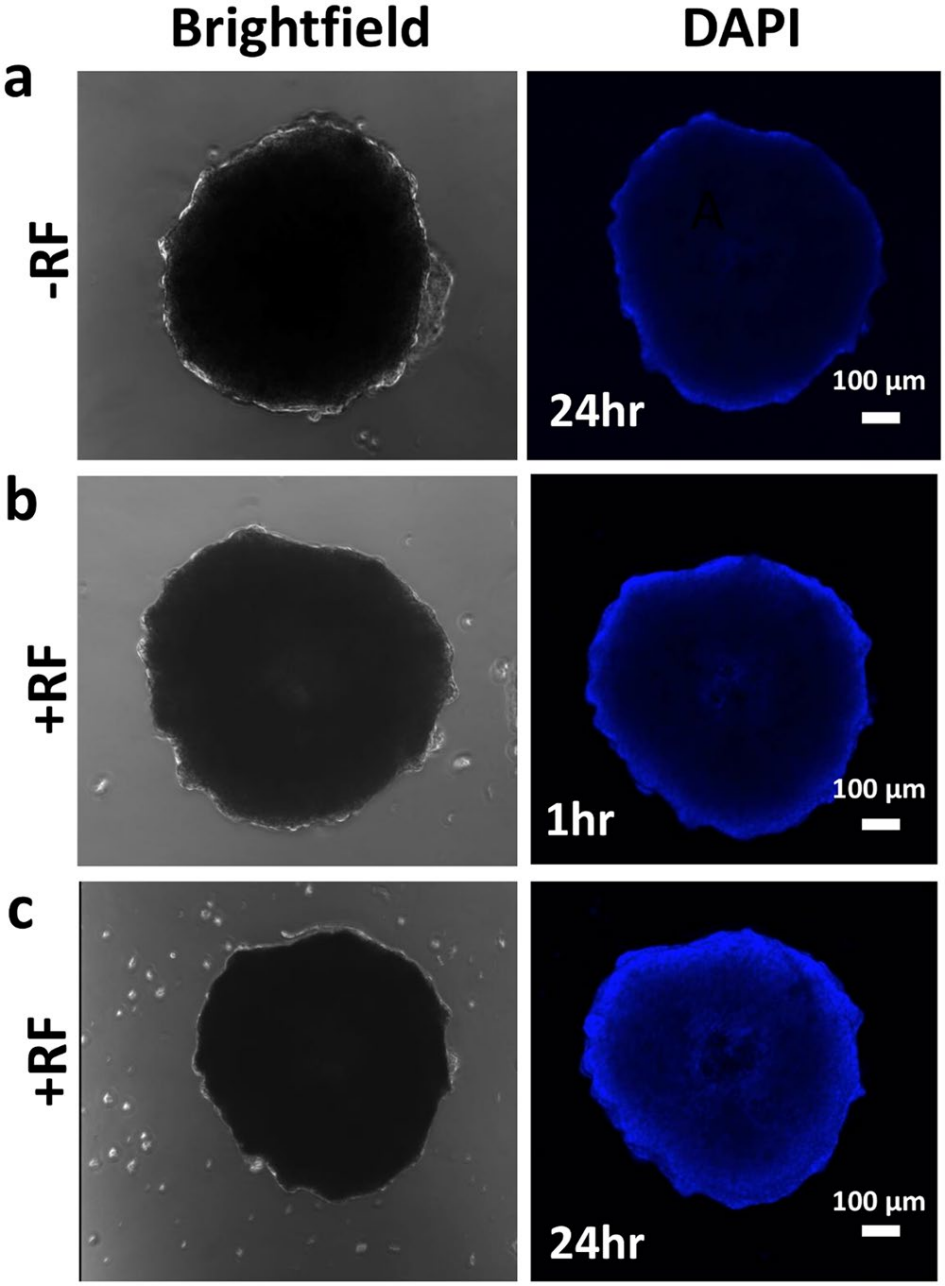
Exposure to non-invasive RF field increases diffusive penetration of molecules in 3D tissue. Left: Brightfield image of untreated Capan-1 spheroid; right: DAPI wavelength image. (**a**) Spheroid untreated with RF and incubated with DAPI for 24 h; (**b**) Spheroid treated with RF and incubated with DAPI for 1 h; (**c**) Spheroid treated with RF and incubated with DAPI for 24 h. Spheroid in (**b**) and (**c**) is the same lesion. Magnification: 10x.

In order to evaluate how RF affects diffusive transport, we first assessed DAPI penetration in PDAC tumor spheroids formed with Capan-1 cells, which were found to form dense structures and, thus, present a high barrier to transport (Fig. 2). DAPI molecular weight (350.3 g/mol) is similar to Gem (299.6 g/mol), both being hydrophilic molecules with 20 and 25 mg/mL water solubility, respectively, and high affinity to the nucleus. Thus, DAPI can be used as a fluorescent surrogate for Gem transport. Results show that the molecules were mostly unable to reach the center of spheroids at 24 h of incubation (Fig. 2c). We further observed that RF alone did not cause significant cell death throughout the 3D structure, but pre-treatment with RF prior to incubation with DAPI/Gem yielded significant increase in DAPI fluorescence through the tissue.

**Figure 3:**
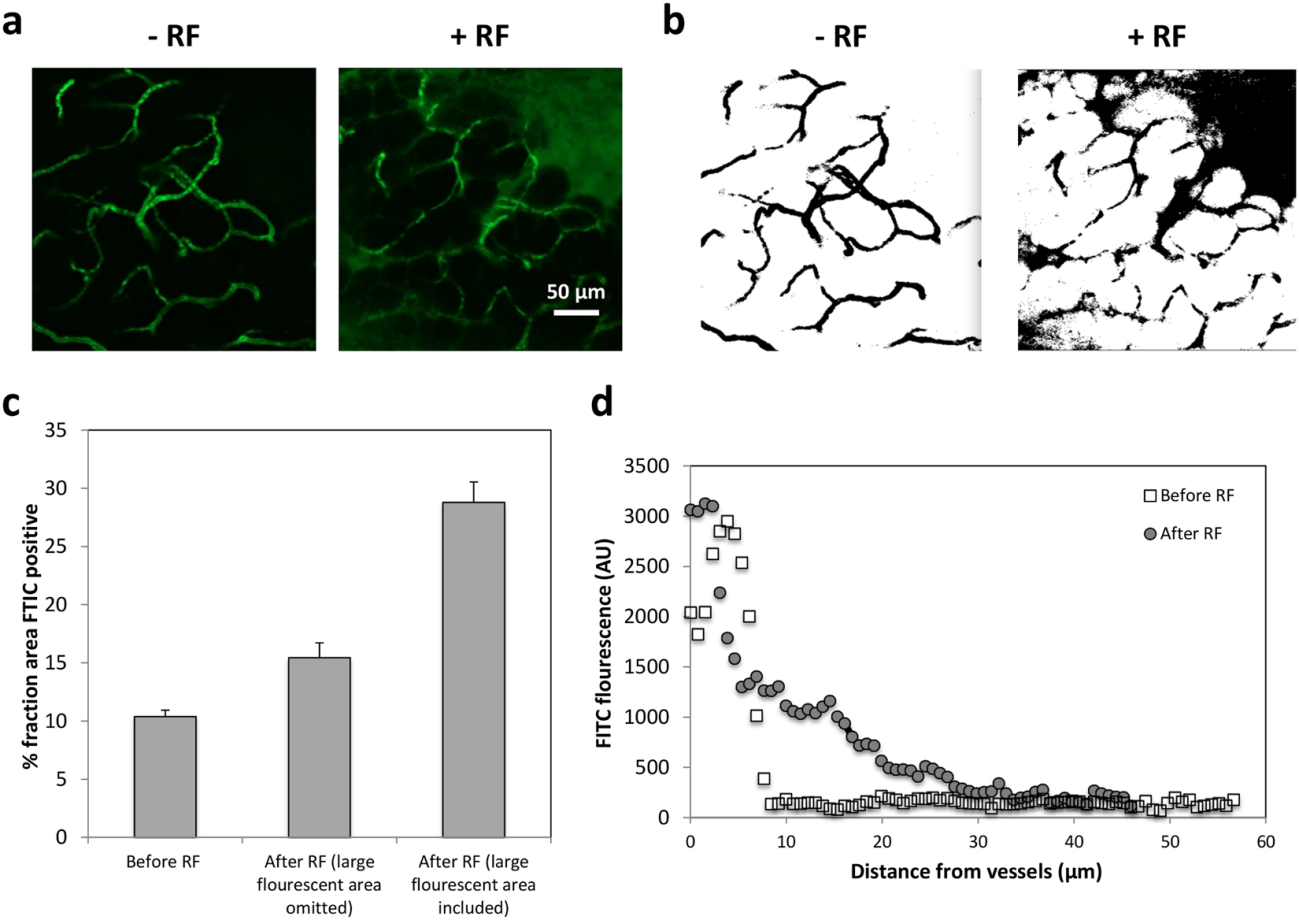
Evaluation of transport in PDAC tumors in mice. (**a**) Representative intravital microscopy images of FITC penetration in tumor tissue following systemic administration before and after RF exposure. (**b**) Sample image analysis to quantify percent area fraction of FITC-positive regions with and without RF, highlighting a large region in the upper right corner (with RF). (**c**) Fraction of tissue area stained for FITC. (**d**) Quantification of distance of FITC-positive regions from the surrounding vessels.

To further evaluate enhanced diffusive transport due to RF, we measured FITC vascular extravasation and diffusion in orthotopic PDAC tumors in mice via intravital microscopy (IVM) (Fig. 3). When compared to unexposed tissue, exposure to RF increased the FITC-positive area by at least 49% (Fig. 3c), with diffusion distances from the vasculature up to twice as large (10 µm vs. 20 µm) (Fig. 3d).

Pancreatic tumors are notorious for being fibrotic, maintaining a high density of extra-cellular matrix (ECM) proteins, which may hinder diffusive transport. To assess the extent of fibrosis, we evaluated the presence of collagen, a major component of the ECM, in stained sections of a human PDAC tissue microarray, finding that the presence of collagen was non-uniform (Supplementary Figure 1). This observation is consistent with our previous work with PDAC 3D cell cultures^24^. We further noted that there was substantial heterogeneity in the amount of overall stroma, with some clinically relevant PDAC tumors exhibiting large regions with little stroma. Although an increased diffusive transport due to RF might help to overcome the transport barrier offered by the typical stroma of PDAC, further study is needed to elucidate the effects of stromal variability on this transport.

### Cell death increases after non-invasive RF plus Gem exposure in 3D cell culture

**Figure 4:**
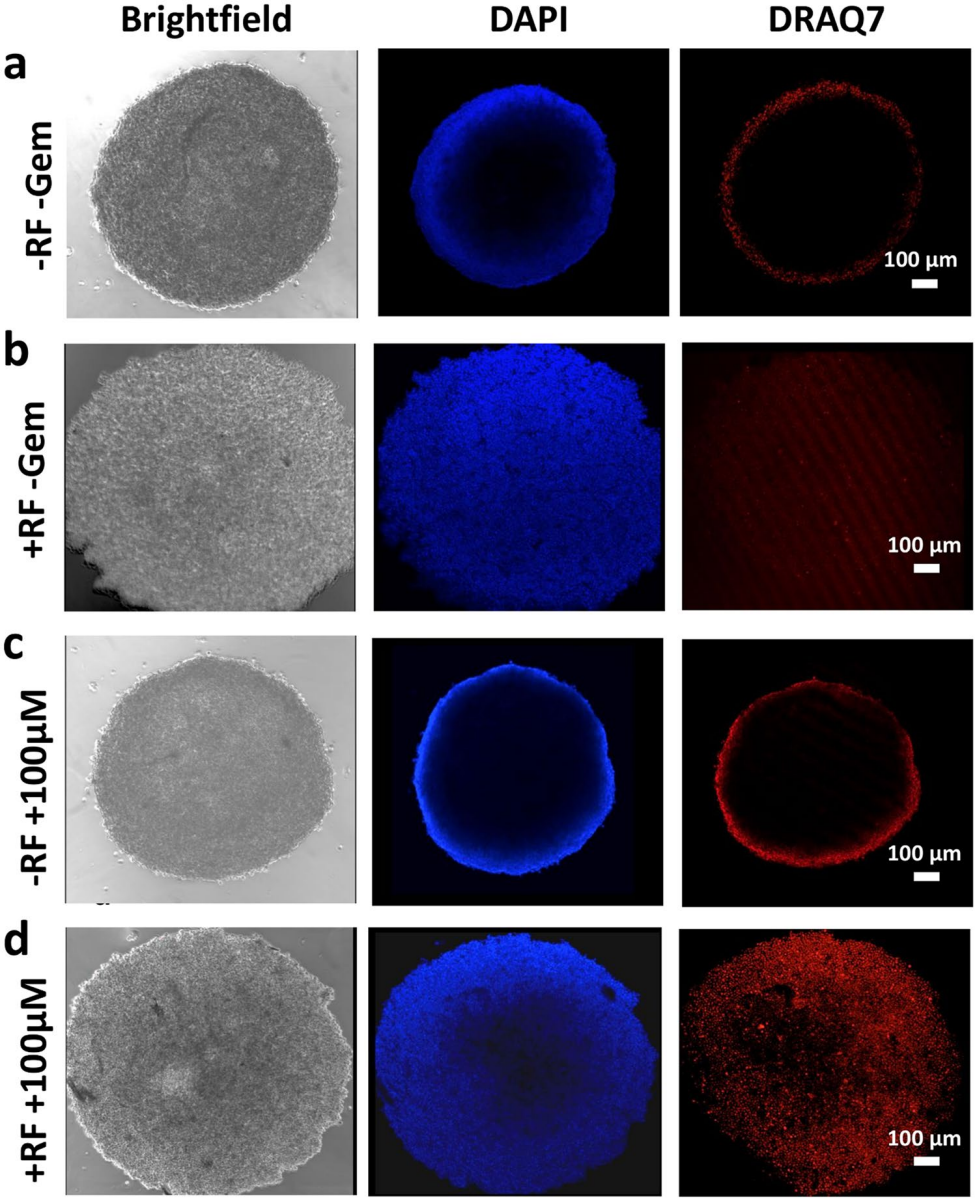
Effect of Gem treatment in non-invasive RF pretreated Capan-1 spheroids. Left to right: Brightfield, DAPI and DRAQ7 channels: (**a**) Untreated spheroid; (**b**) Spheroid exposed to RF only; (**c**) Spheroid exposed to 100 μM Gem only for 24 h; (**d**) Spheroid pretreated with RF and exposed to 100 μM Gem for 24 h.

**Figure 5:**
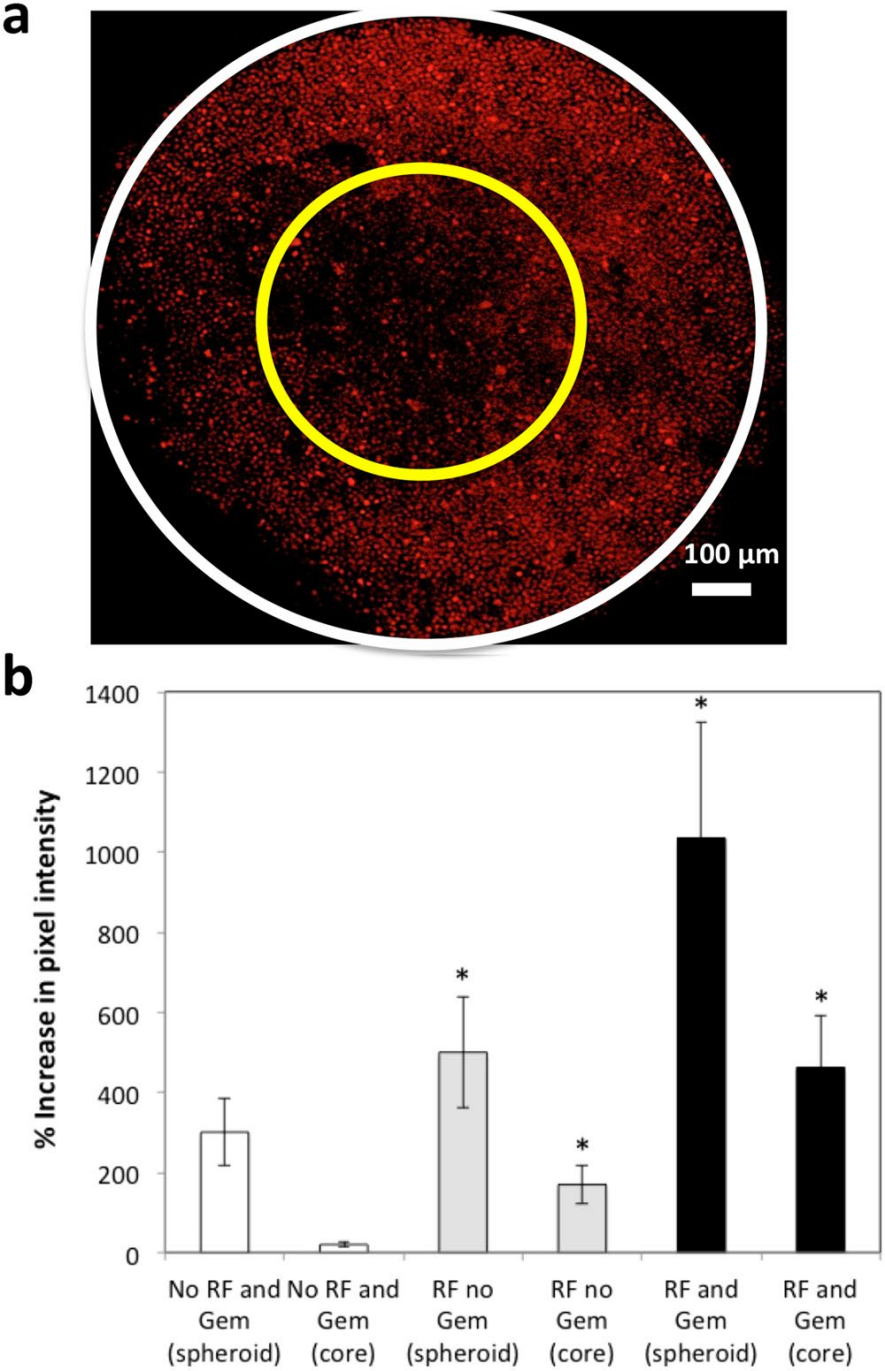
DRAQ7 signals denoting cell death induced by Gem through RF pretreated Capan-1 spheroids. (**a**) A representative spheroid image is shown. For the analysis, the center of the inner circle (enclosing the “core”) was positioned in the center of the spheroid (enclosed by the larger circle), with a radius half that of the whole spheroid. (**a**) Pixel intensity was quantified for whole spheroid (within outer circle) and for spheroid core (within inner circle). The regions of interest (ROI) were randomly selected. (**b**) Percent increase in DRAQ7 intensity for the whole spheroid and for the core, normalized to pixel intensity in the untreated group. Error bars represent standard deviation (n = 3). Statistical significance (*) determined using two-tailed Student’s t-test with significance level of 0.05.

To further examine the effect of RF pretreatment in enabling increased drug penetration in the hypovascularized tumor microenvironment, we incubated 9-day-old Capan-1 spheroids with Gem for 24 h after pretreatment with RF. We used confocal microscopy to acquire Z-stack images through the center of spheroids to evaluate the cell viability, observed via DAPI, and cell death, observed via DRAQ7. Figure 4 shows that RF treatment increased DAPI penetration (Fig. 4b), apparently by increasing cell roundness^10^ and, thus, opening the interstitium for DAPI molecule penetration; the uniformly distributed low DRAQ7 level suggests a corresponding low cytotoxicity. Gem without RF pre-treatment was unable to induce significant cell death (Fig. 4c). In contrast, significant increase in cell death was observed when tumor spheres were treated with Gem immediately after RF treatment (Fig. 4d). Higher staining is noted at the periphery of spheroids not treated with RF (Fig. 4a and c), possibly due to background signal. Figure 5 shows that cell death in the spheroid periphery was 300% higher and in the spheroid core was 1500% higher than for untreated lesions. Interestingly, RF exposure alone yielded 160% increase in cell death on the periphery and 700% increase in the core. Measurement of temperatures using an infrared camera that had previously been calibrated with optical thermal probes, to ensure accurate thermometry, confirmed that the slight temperature rise during RF exposure did not cause undue heating of the cells in 2D or 3D cell culture (Supplementary Figure 2).

### Simulation of heterogeneously hypovascularized tissue

We first simulated an avascular tumor spheroid in cell culture for calibration of the drug effect. The lesion included tumor tissue (proliferating, hypoxic, necrotic) and a dense ECM. Noting that PANC-1 and Capan-1 cells exhibit similar survival at higher Gem concentrations^25^, we matched to data available for the EC70 drug effect for PANC-1 spheroids when treated with 100 μM Gem for 72 h^26^. This drug effect was then used to simulate the response to Gem bolus injection on a lesion *in vivo*. Next, to represent typical hypovascularized PDAC, a tumor with under-developed vascularization was simulated. Heterogeneous ECM concentration simulated fibrosis, which may adversely affect the diffusive transport of drug molecules, tumor angiogenic factors (TAF), and matrix degrading enzymes (MDE) as represented in the model. Supplementary Figure 3 shows a representative simulated hypovascularized lesion prior to treatment evaluation, highlighting the tissue regions (proliferating, hypoxic, necrotic) (A), associated ECM density (B), and corresponding heterogeneous O_2_ concentration profile (C). The proportion of the tissue regions is heterogeneous, with most of the tissue being hypoxic (58%) compared to proliferating (25%) or necrotic (17%).

### Comparison of Gem vs. non-invasive RF plus Gem therapy

**Figure 6:**
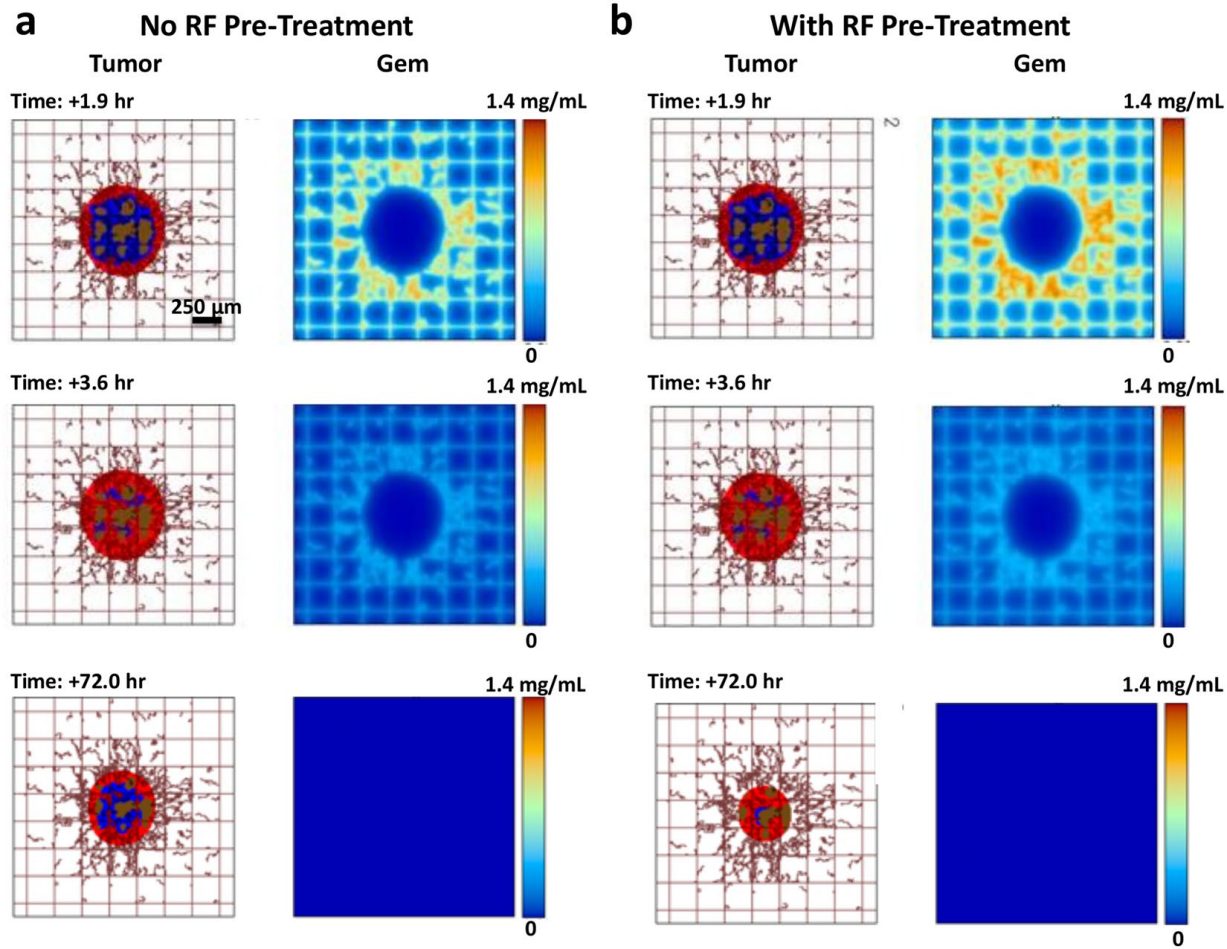
Simulation of combination RF and Gem treatment. (**a**) Gem with no RF pre-treatment; (**b**) Gem with RF pre-treatment. For each panel, vascular flow is from bottom left to upper right. Tumor tissue includes proliferating (red), hypoxic (blue) and necrotic (brown) regions. Existing capillary network is denoted by regularly spaced grid (brown), with vessels induced by angiogenesis shown as irregular lines growing towards the hypoxic tumor regions acting as a source of angiogenic stimuli. At 1.9 hr post injection, the drug concentration is high. Over the course of 3.6 hr, most of the drug washes out. By 72 hr, tumor lesion has visibly shrunk due to the drug effect.

We simulated treatment via bolus injection of drug alone or drug injected after RF pre-treatment. Due to hypovascularization, drug is mainly delivered from the surrounding vessels (Fig. 6), as has been experimentally previously observed^23^. The drug penetration was experimentally measured to be ~20 μm (20% that of O_2_) by 24 h based on a surrogate molecule penetration in Capan-1 spheroids (Fig. 2). As expected, the drug concentration is higher for the RF plus Gem case than for Gem alone, and over the next 2 h the drug washes out of the system. For chemotherapy following RF, the drug concentration follows a similar profile as for drug alone, except with larger magnitude. This is expected, since with RF, the molecule penetration is increased by 75% compared to non-RF (as measured from experimental data, Figs 2 and 4). We also assume that, as a first approximation, the molecule vascular extravasation scales linearly with this increase. During this process, the tumor begins to shrink due to the drug effect, with the regression being more pronounced for the RF plus Gem therapy. Hypoxic regions in the tumor interior transiently switch to proliferation based on tissue death in the tumor periphery opening up access to O_2_ and nutrients from the surrounding vasculature.

### Variation of RF-related effects on drug vascular extravasation and tissue penetration

**Figure 7:**
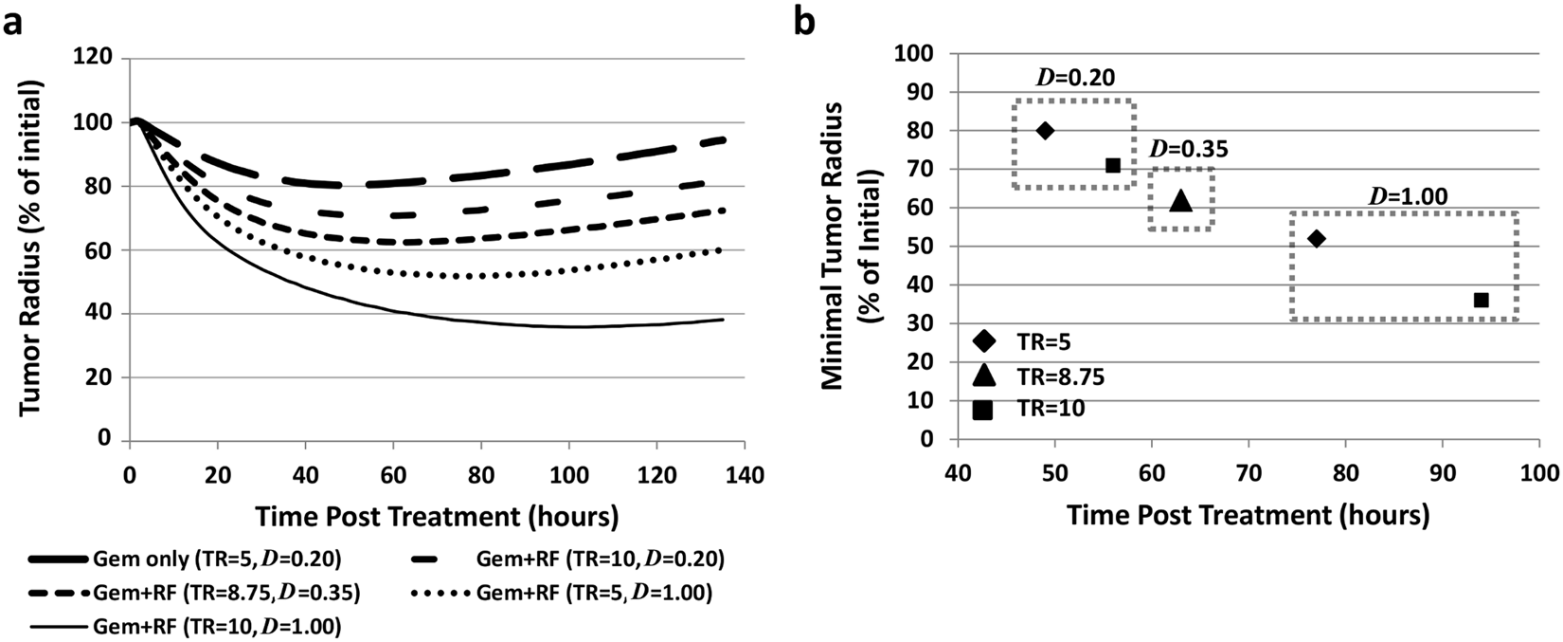
Analysis of treatment simulations. (**a**) Change in simulated tumor lesion radius based on different therapy scenarios. (**b**) Minimal tumor size achieved for each simulated treatment. Dotted boxes enclose cases with like diffusive penetration. A trend of minimal size decreasing in time emerges, which highlights the relative contribution to regression of RF modulation of drug diffusive penetration and vascular extravasation transfer rate. Middle (triangle) indicates Gem penetration and transfer rate increasing by 75% following non-invasive RF exposure (calibrated from experimental data). TR: drug vascular extravasation transfer rate (for O_2_, TR = 5); *D*: drug diffusive penetration (for O_2_, *D* = 1.00).

The response to the simulated therapies is quantified in Fig. 7a. For Gem only, lesion radius is decreased by 19.7% by 48 h post treatment before regrowth resumes, while for Gem following non-invasive RF, for which both vascular extravasation transfer rate (TR) and diffusive penetration (*D*) are increased by 75%, the radius decreases by almost twice as much (36.6% during this timeframe). To further study the potential effect of RF on drug interstitial diffusive transport as well as vascular transfer, we simulated variation in these two key parameters, namely, by maximizing penetration to match O_2_ and doubling the transfer rate. Figure 7a shows that when the TR is doubled but *D* is low (as experimentally measured), therapy performance is better than with Gem alone (29.0% radius decrease by 48 h), but not as good as when both TR and *D* are increased by 75%. When TR is kept the same as for Gem only and *D* is maximized, the lesion radius decreases 44.9% by 48 h. When both drug TR and *D* are maximized, the radius decreases the most (55.3%).

Figure 7b plots the minimal tumor size achieved for each simulated treatment. These data delineate a trend of minimal size decreasing in time highlighting the relative contribution of drug *D* and vascular TR to lesion regression by the RF modulation. The trend shows that variation in *D* elicits a larger change in tumor response than variation in vascular TR. For the same TR, minimal size decreases by 28% to 35% based on penetration ranging from 0.2x to 1x of O_2_, respectively. In contrast, for the same *D*, minimal size decreases by 9% to 16% based on the transfer rate ranging from 1x to 2x that of O_2_, respectively.

### Simulation of repeated therapy for advanced disease

**Figure 8:**
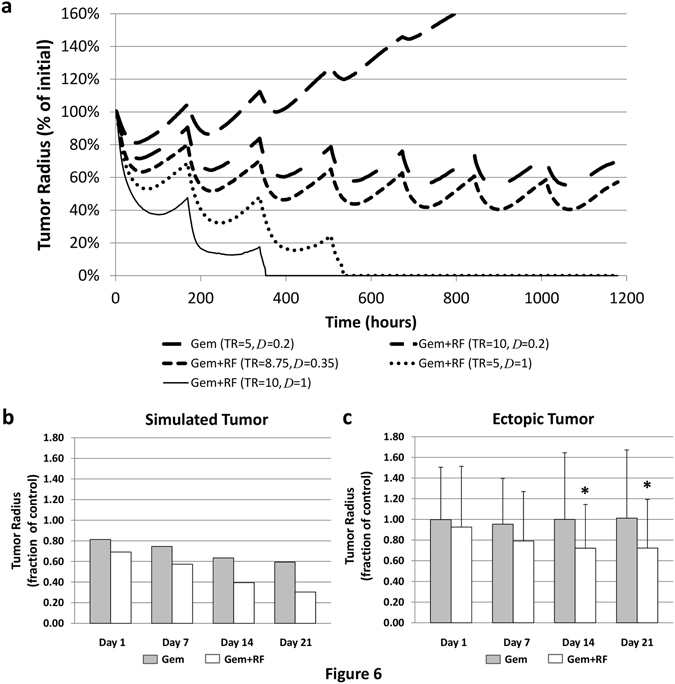
Simulation of weekly repetitive treatment for 7w with Gem or combination of Gem and non-invasive RF. (**a**) For Gem only case (topmost curve, large dash), lesion increases by 166% by the end of 6w compared to the initial size, while for Gem+RF assuming a 75% increase in drug diffusive penetration and vascular extravasation transfer rate (middle curve, short dash; calibrated from experimental data), the lesion is restrained after the 4^th^ treatment. TR: drug extravasation vascular extravasation transfer rate (for O_2_, TR = 5); *D*: drug diffusive penetration (for O_2_, *D* = 1.00). (**b**) Summary of tumor regression as fraction of untreated control predicted by the simulations assuming a 75% increase in drug penetration and transfer rate for Gem+RF. (**c**) Tumor regression measured as fraction of untreated control for PANC-1 ectopic tumors in mice *in vivo* (data from ref. 7). Mean ± SEM. Statistical significance (*)determined using two-tailed Student’s t-test with significance level 0.05.

Next, we simulated weekly treatments for 7w with Gem or combination of Gem and non-invasive RF. This dosing schedule follows current clinical protocol for Stage IV PDAC^27^. Figure 8a shows that Gem only treatment fails to restrain lesion growth, which reaches 166% of initial radius by the end of 6w, and continues unbounded to exceed the limits of the computational domain. Pre-treatment with RF drastically changes this outcome, with the lesion essentially restrained to 60% of initial radius by 4w. Varying the effect of RF on drug diffusive penetration, *D*, and vascular extravasation transfer rate, TR, further highlights the dynamics previously shown for the single treatment scenario (Figs 6 and 7). When the TR is doubled and *D* is kept the same as for the drug only case, the lesion undergoes slow regression over 7w, but it is unclear whether it will eventually be eradicated due to slow buildup of resistance to RF. In contrast, when the TR is unchanged but the drug *D* is maximized (matching O_2_), the lesion is destroyed by 4w. For comparison, a doubled vascular TR in addition to maximized *D* provides a modest advantage, with lesion eradication achieved by 3w. This suggests that the effect of non-invasive RF on drug *D* modulates the tumor response to a larger extent than vascular transfer.

Figure 8b summarizes the tumor radius decrease as a fraction of the untreated control when simulating chemotherapy over the course of 4 treatments once/week for Gem only or Gem+RF. These simulated results follow the trend and fall within the range of tumor regression measurements obtained with PANC-1 ectopic tumors subjected to the same treatment protocols in mice (Fig. 8c), although the simulations suggest an effect from Gem alone that was not experimentally observed. The PANC-1 tumors *in vivo* regressed to 82 ± 33% of untreated control after two treatments^7^, which is consistent with the simulation results. The difference in tumor radius as a fraction of control between the Gem only and Gem+RF cases by 4w was on average 0.28 for the measured (ectopic) tumors and 0.29 for the simulated tumor, a discrepancy of 3%.

## Discussion

We present a novel integration of experimental analysis of pancreatic ductal adenocarcinoma (PDAC) with computational simulation of hypovascularized tumor lesions to evaluate the tissue-scale response to chemotherapy combined with non-invasive RF. PDAC, the fourth leading cause of cancer death in Europe and in the United States^28, 29^, is characterized by hypovascularization, hypoxia, and desmoplasia, and has extremely limited treatment options. A major reason why PDAC is difficult to treat is the extensive stroma and hypovascularization, which could reduce drug transport from the systemic circulation into the tumor microenvironment^30^. Thus, it is imperative to develop new strategies which can promote the transport of anticancer therapeutics into PDAC lesions. To assess the diffusive transport barrier, we evaluated combination therapy of RF and Gem in both 2D (monolayer) and 3D (spheroid) cell cultures. Our previous work has shown that cell-cell adhesion and cell-substrate adhesion are altered after RF to potentially facilitate diffusive transport and molecule vascular extravasation^10^. This is expected to dramatically affect the response to drugs passively diffusing in the tumor microenvironment, as the drug molecules would be able to extravasate in high volume from the vasculature and distribute deeper and more homogeneously throughout the tissue. Further, our experiments (Fig. 4) show that RF enhances PDAC cell death in spheroids representing hypovascularized tumor lesions^31–33^. This suggests that RF influences not only individual cell- but also tissue-scale interactions. The results show that PDAC cells in the spheroid model share key responses observed in monolayer cells when subjected to RF treatment, namely, increased susceptibility due to higher drug uptake, possibly caused by diffusive transport enhanced by cell membrane retraction (see also Supplementary Figure 4).

The monolayer studies further show that non-invasive RF causes immediate sensitivity to Gem in PDAC cells (Fig. 1), which may additionally explain the enhanced cell death in the 3D cell culture when subjected to RF plus Gem (Fig. 4). The cytostatic effect observed in pancreatic cancer cells with RF plus Gem was previously observed and attributed to induction of autophagy when cells were exposed to 13.56 MHz radio waves^7^. It was found that excessive numbers of autophagosomes in cancer cells persisted during 24 h to 48 h after RF exposure and then declined. The addition of a sub-toxic dose of Gem to RF treatment inhibited the cell recovery from the RF-induced autophagy and enhanced cytotoxicity. When systemic wide responses were investigated, it was further observed that treatment of pancreatic cancer *in vivo* in mice with a combination of non-invasive RF and Gem had superior antitumor effect compared to RF or Gem alone^7^. We expect these findings to aid in the development of efficient non-invasive RF and Gem treatment schedules for potential clinical application. To this end, we simulated the tissue-scale response via computational modeling, showing that drug diffusive transport may be a key parameter determining overall system behavior (Fig. 7). Assessment of a hypothetical schedule of non-invasive RF combined with Gem following the current clinical protocol to treat Stage IV PDAC further suggests that growth of lesions unresponsive with Gem alone may be restrained when exposed to RF and Gem combination (Fig. 8). This prediction is compared with experimental observations of PANC-1 tumors grown in mice *in vivo* ^7^. The simulations project tumor regression towards the low end of the experimental measurements, suggesting that additional factors not included in the mathematical model may also present resistance to the treatment *in vivo*. Future modeling work will further evaluate the parameters associated with *in vivo* performance of Gem therapy combined with RF.

Certain PDAC cell sub-populations displayed evidence of a developed ability to become less susceptible to RF over time. This was characterized by negligible membrane retraction in response to RF after a single exposure. Moreover, multiple RF exposures seemed to sort sub-populations by their susceptibility to non-invasive RF or by their innate behaviors in response to treatment, as there were some cells within the population which always rounded and detached from the substrate and never developed lowered susceptibility. This finding was unexpected and suggests the preexistence of cell sub-populations that are more RF-sensitive and, therefore, more readily detachable from the substrate. There may also be emergent sub-populations, displaying increased phenotypic plasticity by developing an ability to compensate for the diverse effects of RF, remaining adhered to the substrate surface and surviving even after multiple treatments. These findings may help to provide insight into the mechanisms involved in cancer cell sensitivity to RF as well as the rational design of appropriate treatment schedules in combination with chemo- or radiation therapy. Further elucidation of these phenomena would also influence the possibility to fine-tune therapy parameters for maximizing tumor regression. In particular, a more detailed quantification of the loss of susceptibility of PDAC to RF in consequent exposures would enable a more comprehensive evaluation of tumor response via the computational modeling.

The RF-facilitated drug transport in the tumor microenvironment due to transient cell phenotypic changes would be complementary to any enhancement of transport due to alleviation of the diffusion barrier presented by fibrotic ECM. Recent work has linked ECM-induced swelling stress in orthotopic pancreatic tumors to decreased vascular perfusion^34^. Approaches that aim to “thin out” the ECM components have been proposed to enhance the transport of chemotherapeutic drug molecules diffusing out of the vasculature. For example, inhibition of angiotensin has been found to inhibit stromal collagen and hyaluronan production in an orthotopic pancreatic cancer model, which decreased solid stress and evinced increased vascular perfusion, lower hypoxia, and improved drug delivery^35^. It is unclear, however, to what extent angiotensin inhibition by itself affects tumor progression. A recent population-based cohort study failed to show a decreased risk of pancreatic cancer through the use of angiotensin-converting enzyme inhibitors or angiotensin receptor blockers^36^, even though such inhibitors have been shown to suppress pancreatic cancer growth in pre-clinical models^35, 37, 38^.

This study presents an interdisciplinary approach linking the cell- to the tissue-scale to evaluate potential response to combination therapy of non-invasive RF and Gem for PDAC. The proposed approach enables the study of tumor-specific conditions hindering therapy effectiveness, such as diffusive transport limitations resulting from tissue hypovascularization. The modulation of drug diffusive penetration and vascular extravasation transfer rate by RF allows relating the treatment parameters to drug-specific diffusive penetration and vascular extravasation characteristics. The integration of experiments with computational modeling offers the possibility to fine-tune these parameters to maximize tumor regression while avoiding the disadvantages of under- or over-treatment. Longer term, the approach may further enable assessment of potential clinical protocols to evaluate therapy response based on patient tumor-specific information, integrating data from both experiments and simulations using cell and tissue information obtained from imaging and biopsy.

## Methods

### Cell lines

Human pancreatic PDAC cancer cell lines Capan-1 and PANC-1 (American Type Culture Collection) were maintained in Iscove’s Modified Dulbecco’s Media (IMDM) with 4 mM L-glutamine and 20% Fetal Bovine Serum (FBS), and in Dulbecco’s Modified Eagle’s Medium (DMEM) with 10% FBS, respectively, both supplemented with penicillin 2% and cultured in 5% CO2 at 37 °C.

### Three-dimensional cell culture

Spheroids were created using a novel approach we recently reported combining two traditional techniques, namely, hanging drop and methylcellulose in the medium^39^. Briefly, the specific cell type base medium, containing 10% FBS and supplemented with methylcellulose stock solution in 80:20 ratio, respectively, was used. Methylcellulose stock solution was prepared by dissolving autoclaved methylcellulose (6 g) powder (M0512, Sigma-Aldrich) in 250 mL preheated to 60 °C basal medium for 20 min. Thereafter, 250 mL medium (at room temperature, RT) containing FBS (20%) was added to final volume of 500 mL and the whole solution mixed overnight at 4 °C. Final stock solution was aliquoted and cleared by centrifugation (5000 g, 2 h, RT). Only the clear, highly viscous supernatant was used for the spheroid assay (about 90–95% of stock solution). For spheroid generation, 20% of stock solution and 80% culture medium corresponding to final 0.24% methylcellulose were used. 20 μL drops containing 20,000 cells were pipetted onto the lid of 100 mm^2^ dishes and inverted over dishes containing 10 mL phosphate buffer solution (PBS). Hanging drop cultures were incubated under standard culture conditions for 1week, which provided time for cell sedimentation. Each spheroid was gently caught by sterile spatula and transferred to a well (12 well plates) for treatment or imaging.

### RF treatment and evaluation of cell heating

For experiments in two-dimensional (2D) cultures, 200,000 cells per well were seeded in 12 well plates. Twenty four hours later, when attachment had occurred, the plates were placed in non-invasive RF generator^40^ and exposed to RF (900 W, 13.56 MHz, RT) for 5 min. Three-dimensional (3D) cell cultures were similarly exposed for 5 min to RF. RF power absorption was calibrated by temperature, recorded in real time via infrared thermography (FLIR Systems Inc., Boston, MA) to ensure a consistent field across wells within the plate and over time. For cells undergoing multiple RF treatments, a small amount of media was added every two days but not replaced so that non-adhered cell sub-population, residing in the supernatant, would be preserved. Parameters were measured using brightfield images, obtained using ImageXpress (Molecular Devices, CA, USA). No fluorescent probes were used to avoid disturbing cell response to RF. In order to maintain consistent temperatures, treatment groups were kept in the incubator (37 °C) until RF treatment. Following treatment, the cell plates were transferred to the incubator for maintenance of temperature. Controls were taken out of the incubator and their temperature was allowed to drop to 34 °C (similar to the RF treatment group) before placing them back in the incubator for maintenance of temperature.

### Cytotoxicity analysis

Cytotoxicity was quantified using DRAQ7 (1,5-Bis{[2-(dimethylamino)ethyl]amino}-4,8-dihydroxyanthracene-9,10-dione) staining, which is a far-red fluorescent dye, kindly provided by Biostatus Ltd (UK). DRAQ7 is excluded from viable cells and hence only stains nuclei in dead and/or cell membrane permeabilized cells, as it passively diffuses through the damaged cytoplasmic and nuclear membranes and eventually binds with A-T rich repeats in DNA. DRAQ7 has peak absorbance at 600 nm and 646 nm and fluoresces in the far-red/near infra-red peaking at 697 nm (when bound to double stranded DNA). Cells were seeded at a concentration of 70,000 cells per well in Greiner Bio One Cellstar® tissue culture 6-well plate for 24 h, washed twice in serum-free medium and incubated with 2 mL of complete medium containing 3 µM DRAQ7. High throughput, time-lapse based experiments were performed within 5–10 min of dosing using the In-Cell Analyzer 2000 microscope (GE Healthcare, UK), as previously described^41^. Briefly, images were obtained hourly for 24 h. Over 4,000 cells were imaged at each timepoint at 20x magnification in DRAQ7 and brightfield channels. After the time-lapse the cells were dosed with very high dose (10 μM) of polyethylenimine coated QDs in 2 mL media with 0.5 h exposure, which killed 100% of cells due to high toxicity. Additional set of images was collected from which total number of cells in each well was obtained. Death was quantified by automated measure of DRAQ7 signals at each time point using In-Cell Developer Toolbox software (GE Healthcare, UK) and was displayed as % of total cells within each well. Additionally, separate time-lapse analysis was performed from 0–43 h to confirm stability of DRAQ7 signals. An automated algorithm segmented the DRAQ7 nuclear signals by first recognizing pixels that displayed intensity ≥ 750grey levels above background intensity. Secondly, signal was gated via pixel area (100pixels^2^ < DRAQ7 segmented signal < 1000pixels^2^) to discount anomalous components. Signals which overlapped frame edges were discounted.

### Cell phenotype analysis

Cell membrane retraction was analyzed using the method we previously described^10^. Briefly, PDAC cells were grown to ~70% confluence and ImageXpress high-throughput microscope was used to acquire brightfield images (x20 magnification) before and immediately after RF hourly for 24 h. MATLAB software (Mathworks, USA) was used to create a brightfield textural analysis algorithm^10^ to quantify the space in the frame not occupied by cells, and this area was normalized to the space between cells before the RF exposure. Manual segmentation of brightfield images before, 0 and 2 h after RF was performed by highlighting the cell perimeter using ImageJ 1.47 Software (National Institutes of Health, USA) and measuring area within the selection. Over 600 cells were measured per group in random 10–15 fields for statistically robust results that considered whole cell populations. Image analysis was repeated to measure response, i.e., cell area and hence membrane retraction, of single cells on surface of spheroids. Membrane retracted cells were those which displayed at least a 33% decrease in cell area.

### Evaluation of transport

Tumor spheroids were pretreated with RF, and DAPI (4’,6-Diamidino-2-Phenylindole) (3 µM) was immediately added. After specific time period was reached (0–72 hours), spheroids were fixed in 4% paraformaldehyde. DAPI was used as a fluorescent probe resembling hydrophilic and low molecular weight Gem. Similar to Gem, it also has high affinity for nucleic acids. DAPI penetration was measured (n = 6) by evaluating the staining intensity from the periphery into the spheroid core, and normalized by the outermost (boundary) value to arrive at an average fractional value. DAPI alone did not evince effects on the spheroid growth.

### Protocol for *in vivo* experiments

The *in vivo* experiments were approved by the Institutional Animal Care and Use Committee (IACUC) at the Baylor College of Medicine (No. AN-6448). SCID mice (4–6 weeks of age) were purchased from the National Cancer Institute Mouse Repository, and maintained under pathogen-free conditions and treated in accordance with the recommendations in the National Institute of Health’s Guide for the Care and Use of Laboratory Animals. Mice were sedated and grounded to receiving plates with conducting copper tape to prevent thermal injury as previously described^8^. The mice were exposed weekly to RF (600 W, 13.56 MHz, RT, 10 min) and each experiment was repeated 3 times.

### Orthotopic tumor model

Mice (10–13 per group) were injected with 2–3 × 10^6^ PDAC cells suspended in 40 μL of Matrigel directly into the pancreas with concurrent abdominal IVM window placement over the pancreas^42^. After 1 week of incubation, the pancreas was imaged via IVM through the abdominal window. 100 µL of FITC dextran (70 k MW, 5 mg/mL; Molecular Probes, Eugene, Oregon) was injected via the retro-orbit to visualize the vasculature. After a delay of 2.5 hours, RF was applied to the left abdominal region to a target surface temperature of 40 °C for 20 min. The vasculature post-RF was re-imaged via IVM with another injection of 100 µL FITC dextran.

### Ectopic tumor model

The data for the ectopic tumor model was previously obtained as described in ref. 7. Briefly, mice (10–13 per group) were injected with 1–3 × 10^6^ PDAC cells subcutaneously, and tumors were measured by width, length, and height, with tumor volume calculated as width x length x height. Animals started treatment when average tumor volume reached 100 mm^3^. Gem dose of 70 mg/kg was given intraperitoneally, and the tumors were exposed the next day to non-invasive RF for 10 min.

### Human PDAC histology

To evaluate the collagen content in clinical samples (T142 Pancreatic cancer tissue array, Biomax, USA), hematoxylin and eosin (H&E) as well as collagen staining (Picro Sirius Red Staining Kit, Abcam, USA) were performed on pathologically diagnosed separate samples in the tissue microarray. All tissues in the repository are collected with donor consent under HIPPA approved protocols.

### Simulation of tumor response to RF and drug therapies

The computational model (see Supplementary information for details) builds upon previous work^43–46^ to simulate the effects of RF combined with drug transport impaired by hypovascularization and extra-cellular matrix. The model parameters (Supplementary Table 1) were determined from experimental data as described in Results and Supplementary information. Simulations were run to evaluate the response to the administration of Gem as bolus injection over the course of 5w. A control (untreated) case was also simulated in order to compare the tumor responses. To simulate therapy for advanced metastatic disease (Stage IV)^27^, the drug administration was simulated once weekly for 7w.

### Statistics

Statistical significance shown in the results was determined using two-tailed Student’s t-test with significance level 0.05 (*) or 0.01 (**) using GraphPad Prism (GraphPad Software).

### Data Availability

All data generated or analyzed during this study are included in this published article and its Supplementary Information files.

## Electronic supplementary material

Supplementary  information

## References

[1] Chauhan VP, Stylianopoulos T, Boucher Y, Jain RK (2011). Delivery of molecular and nanoscale medicine to tumors: transport barriers and strategies. Annu. Rev. Chem. Biomol. Eng..

[2] Kleeff J (2007). Pancreatic cancer microenvironment. Int. J. of Cancer.

[3] Koay EJ (2014). Intra-tumoral heterogeneity of gemcitabine delivery and mass transport in human pancreatic cancer. Phys. Biol..

[4] Minchinton AI, Tannock IF (2006). Drug penetration in solid tumours. Nat. Rev. Cancer.

[5] Loehrer PJ (2011). Gemcitabine alone versus gemcitabine plus radiotherapy in patients with locally advanced pancreatic cancer: an Eastern Cooperative Oncology Group trial. J. Clin. Oncol..

[6] Huang J (2011). Long-term results of full-dose gemcitabine with radiation therapy compared to 5-fluorouracil with radiation therapy for locally advanced pancreas cancer. Radiother. Oncol..

[7] Koshkina NV, Briggs K, Palalon F, Curley SA (2014). Autophagy and enhanced chemosensitivity in experimental pancreatic cancers induced by noninvasive radiofrequency field treatment. Cancer.

[8] Glazer ES (2010). Noninvasive Radiofrequency Field Destruction of Pancreatic Adenocarcinoma Xenografts Treated with Targeted Gold Nanoparticles. Clin. Cancer Res. : an official journal of the American Association for Cancer Research.

[9] Raoof M (2013). Tumor selective hyperthermia induced by short-wave capacitively-coupled RF electric-fields. PLoS ONE.

[10] Ware MJ (2015). Radiofrequency treatment alters cancer cell phenotype. Sci. Rep.

[11] Corr SJ (2015). A New Imaging Platform for Visualizing Biological Effects of Non-Invasive Radiofrequency Electric-Field Cancer Hyperthermia. PLoS ONE.

[12] van der Zee J (2002). Heating the patient: a promising approach?. Ann. Oncol..

[13] Owusu RA, Abern MR, Inman BA (2013). Hyperthermia as adjunct to intravesical chemotherapy for bladder cancer. Biomed. Res. Int.

[14] Franckena M, van der Zee J (2010). Use of combined radiation and hyperthermia for gynecological cancer. Curr. Opin. Obstet. Gynecol..

[15] Curley SA, Palalon F, Sanders KE, Koshkina NV (2014). The effects of non-invasive radiofrequency treatment and hyperthermia on malignant and nonmalignant cells. Int J Environ Res. Public Health.

[16] Raoof, M. *et al*. Hyperthermia inhibits recombination repair of gemcitabine-stalled replication forks. *J. Natl. Cancer Inst*. **106**, (2014). 10.1093/jnci/dju183 PMC4155430 25128695

[17] Curley SA, Palalon F, Lu X, Koshkina NV (2014). Noninvasive radiofrequency treatment effect on mitochondria in pancreatic cancer cells. Cancer.

[18] Pandya GJ, Shelat VG (2015). Radiofrequency ablation of pancreatic ductal adenocarcinoma: The past, the present and the future. World J. Gastrointest. Oncol.

[19] Matsui Y (2000). Selective thermocoagulation of unresectable pancreatic cancers by using radiofrequency capacitive heating. Pancreas.

[20] El-Serag HB (2004). Hepatocellular carcinoma: recent trends in the United States. Gastroenterology.

[21] Levenback BJ, Sehgal CM, Wood AK (2012). Modeling of thermal effects in antivascular ultrasound therapy. J. Acoust. Soc. Am..

[22] Waterman FM, Tupchong L, Matthews J, Nerlinger R (1989). Mechanisms of heat removal during local hyperthermia. Int. J. Radiat. Oncol. Biol. Phys..

[23] Huxham LA, Kyle AH, Baker JH, Nykilchuk LK, Minchinton AI (2004). Microregional effects of gemcitabine in HCT-116 xenografts. Cancer Res..

[24] Ware MJ (2016). Generation of an *in vitro* 3D PDAC stroma rich spheroid model. Biomaterials.

[25] Giovannetti E, Mey V, Danesi R, Mosca I, Del Tacca M (2004). Synergistic cytotoxicity and pharmacogenetics of gemcitabine and pemetrexed combination in pancreatic cancer cell lines. Clin. Cancer Res.: an official journal of the American Association for Cancer Research.

[26] Wen Z (2013). A spheroid-based 3-D culture model for pancreatic cancer drug testing, using the acid phosphatase assay. Braz. J. Med. Biol. Res..

[27] Burris HA (1997). Improvements in survival and clinical benefit with gemcitabine as first-line therapy for patients with advanced pancreas cancer: a randomized trial. J. Clin. Oncol..

[28] Malvezzi M (2015). European cancer mortality predictions for the year 2015: does lung cancer have the highest death rate in EU women?. Ann. Oncol.

[29] American Cancer Society at www.cancer.org. Accessed 3.27.2017.

[30] Erkan M (2012). The role of stroma in pancreatic cancer: diagnostic and therapeutic implications. Nat. Rev. Gastroenterol. Hepatol.

[31] Desoize B (2000). Contribution of three-dimensional culture to cancer research. Crit. Rev. Oncol. Hematol.

[32] Kunz-Schughart LA (1999). Multicellular tumor spheroids: intermediates between monolayer culture and *in vivo* tumor. Cell Biol. Int..

[33] Hirschhaeuser F (2010). Multicellular tumor spheroids: An underestimated tool is catching up again. J. Biotech..

[34] Voutouri C, Polydorou C, Papageorgis P, Gkretsi V, Stylianopoulos T (2016). Hyaluronan-Derived Swelling of Solid Tumors, the Contribution of Collagen and Cancer Cells, and Implications for Cancer Therapy. Neoplasia.

[35] Chauhan VP (2013). Angiotensin inhibition enhances drug delivery and potentiates chemotherapy by decompressing tumour blood vessels. Nat. Commun..

[36] Mandilaras V, Bouganim N, Yin H, Asselah J, Azoulay L (2017). The use of drugs acting on the renin-angiotensin system and the incidence of pancreatic cancer. Br. J. Cancer.

[37] Fujimoto Y, Sasaki T, Tsuchida A, Chayama K (2001). Angiotensin II type 1 receptor expression in human pancreatic cancer and growth inhibition by angiotensin II type 1 receptor antagonist. FEBS Lett.

[38] Arafat HA (2007). Antihypertensives as novel antineoplastics: angiotensin-I-converting enzyme inhibitors and angiotensin II type 1 receptor blockers in pancreatic ductal adenocarcinoma. J. Am. Coll. Surg.

[39] Ware, M. J. *et al*. Generation of homogeneous 3D pancreatic cancer cell spheroids using an improved hanging drop technique. *Tissue Eng. C*, (2016) in press. 10.1089/ten.tec.2015.0280 PMC4827286 26830354

[40] Corr, S., Raoof, M., Wilson, L. & Curley, S. In *ACS Symposium Series* Vol. 1113 Ch. 6, 81–94 (American Chemical Society, 2012).

[41] Ware MJ (2014). Analysis of the influence of cell heterogeneity on nanoparticle dose response. ACS Nano.

[42] Ritsma L (2013). Surgical implantation of an abdominal imaging window for intravital microscopy. Nat. Protoc..

[43] Wu M (2013). The effect of interstitial pressure on tumor growth: Coupling with the blood and lymphatic vascular systems. J. Theor. Biol..

[44] van de Ven AL (2012). Integrated intravital microscopy and mathematical modeling to optimize nanotherapeutics delivery to tumors. AIP Adv.

[45] Wu M (2014). The effect of interstitial pressure on therapeutic agent transport: Coupling with the tumor blood and lymphatic vascular systems. J. Theor. Biol..

[46] Curtis LT, England CG, Wu M, Lowengrub J, Frieboes HB (2016). An interdisciplinary computational/experimental approach to evaluate drug-loaded gold nanoparticle tumor cytotoxicity. Nanomedicine (Lond.).

