# Supplementary material for: Pancreatic adenocarcinoma response to chemotherapy enhanced with non-invasive radio frequency evaluated via an integrated experimental/computational approach
Source: Sci Rep. 2017 Jun 13;7:3437. doi: 10.1038/s41598-017-03040-0 (PMC5469743; doi:10.1038/s41598-017-03040-0)
Supplement: Supplementary file 1 — Supplementary information [file 41598_2017_3040_MOESM1_ESM.pdf]

SUPPLEMENTARY MATERIAL FOR:

Pancreatic adenocarcinoma response to chemotherapy enhanced with non-invasive radio frequency evaluated via an integrated experimental/computational approach

Matthew J. Ware[1,§], Louis T. Curtis[2,§], Min Wu[3], Jason C. Ho[1], Stuart J. Corr[1,4,5], Steven A. Curley[1], Biana Godin[6,*], Hermann B. Frieboes[2,7,*]

[1] Department of Surgery, Baylor College of Medicine, Houston, TX, USA
[2] Department of Bioengineering, University of Louisville, Louisville, KY, USA
[3] Department of Engineering Sciences and Applied Mathematics, Northwestern University, Chicago, IL, USA
[4] Department of Chemistry and Smalley-Curl Institute, Rice University, Houston, TX, USA
[5] Department of Bioengineering, University of Houston, Houston, TX, USA
[6] Department of Nanomedicine, Houston Methodist Research Institute, Houston, TX, USA
[7] James Graham Brown Cancer Center, University of Louisville, Louisville, KY, USA

§ Joint first authorship
*Joint senior authorship

CORRESPONDENCE:

Biana Godin: Department of Nanomedicine, Houston Methodist Research Institute, 6670 Bertner Ave., R8-213, Houston, TX 77030, USA. Phone: 713-441-7329; E-mail: bgodin@houstonmethodist.org; bianagodinv@gmail.com

Hermann B. Frieboes: Department of Bioengineering, Lutz Hall 419, Louisville, KY 40208, USA. Phone: 502-852-3302; Fax: 502-852-6802; E-mail: hbfrie01@louisville.edu

Calibration of RF power absorption and tumor spheroid formation

The RF power absorption by cells in culture was calibrated by temperature, as described in **Methods**, to ensure a consistent field across the wells within the plate and in time over a 10 ^{o}C range in temperature (**Supplementary Figure 5**). The temperature increased by 0.32°C (SD 1.11) within the first minute of exposure and displayed a linear heating rate of 1.38°C min^{-1} (SD = 0.23) from 1 min to 5 min. The final temperature was 38.68°C (SD=0.36) after 5 min. These results were consistent in serum containing DMEM and serum free KSFM media.

Tissue morphology changes after RF exposure in 3D cell culture (tumor spheroids)

We used a highly reproducible and easy to perform hanging drop method based on the addition of methylcellulose for formation of tumor spheroids representing hypo-vascularized PDAC lesions. The resulting 3D cell cultures are uniform in nature and do not easily dissemble when handled, as they are highly rounded with a smooth and defined boundary that allows for increased reproducibility and scalability [1]. We examined morphological changes caused by RF to the 3D tissue structures via scanning electron microscopy (SEM) before and after RF treatment. When treated with RF, cells are seen detaching from the spheroid surfaces over 24h (**Supplementary Figure 4**), as previously observed in monolayer [2]. This detachment decreases the overall spheroid size while providing a possible mechanism for enhanced drug diffusion, and leads to "smoother" looking spheroid surfaces.

Mathematical modeling of tumor response to RF and drug therapies

Hypoxic, necrotic, and viable tumor tissue are represented in a 2D Cartesian coordinate system in which the initial condition is a small lesion (~50 µm diameter) surrounded by a capillary grid of pre-existing vessels. Equations for mass conservation describe tissue growth due to proliferation as a function of access to nutrients and tissue death due to low oxygen (hypoxia) or therapy. These equations are combined with diffusion of small molecules, including oxygen, cell nutrients, drug, tumor angiogenic factors and matrix degrading enzymes, to a reaction-diffusion equation. Spatio-temporally heterogeneous rate constants for death and proliferation are modulated by the availability of oxygen,

nutrients, and drug. The main parameters of the model are described in **Supplementary Table 1**. Details of the numerical implementation are included in [3] and associated references.

Tumor growth

The tumor component is based on [4]. Briefly, the tumor tissue is denoted by Ω and its boundary by Σ. The tumor tissue may have a proliferating region Ω_P in which cells have sufficient oxygen and nutrients, a hypoxic region Ω_H in which oxygen and nutrients are sufficient for survival but not for proliferation, and a necrotic region Ω_N in which oxygen and nutrients are insufficient for survival. The tumor growth velocity (non-dimensionalized) is implemented via a generalized Darcy's law [4]:

$$\mathbf{v}_c = -\mu \nabla P + \chi_E \nabla E \tag{1}$$

where μ is cell-mobility representing the net effects of cell-cell and cell-matrix adhesion, P is oncotic pressure, χ_E is haptotaxis, and E is ECM density. Definitions for χ_E and E are in [4]. By assuming that the cell density is constant in the proliferating region, the overall tumor growth can be associated with the rate of volume change:

$$\nabla \cdot \mathbf{v}_c = \lambda_p \tag{2}$$

where λ_p is the non-dimensional net proliferation rate (see below).

Angiogenesis

The angiogenesis component simulates the model by [5] and is based on [3,6], representing blood flow, vascular leakage and vascular network remodeling due to wall shear stress and mechanical stresses imposed by the tumor tissue. The angiogenesis model is described in detail in [3,6]. As the tumor grows within the vascular environment, the tissue experiences heterogeneous access to elements diffusing from the vasculature, which may depend on tissue pressure, ECM density, and distance from the nearest vascular source. The angiogenesis is modulated by tumor angiogenic factors (TAF) that are released by the hypoxic tissue [3,6]:

$$0=\nabla\cdot\left(D_T\nabla T\right)+\overline{\lambda}^{T}_{production}(1-T)\mathbf{1}_{\Omega_H}-\overline{\lambda}^{T}_{binding}\mathbf{1}_{vessel}-\overline{\lambda}^{T}_{ECM.uptake}ET-\overline{\lambda}^{T}_{decay}T \qquad (3)$$

The TAF diffuse through the tissue with coefficient D_T, are produced in the tumor hypoxic region with a rate $\overline{\lambda}^{T}_{production}$, bind to the surrounding capillary vessels with a rate $\overline{\lambda}^{T}_{binding}$, are uptaken by the ECM (with density E, see below) with a rate $\overline{\lambda}^{T}_{ECM.uptake}$, and decay with a rate $\overline{\lambda}^{T}_{decay}$.

Transport of oxygen and nutrients

The transport of oxygen and nutrients (collectively denoted by σ) through tumor tissue is simulated from the location of extravasation from the vasculature. Oxygen and nutrients are supplied from the neo- and pre-existing vasculature with extravasation rates $\lambda^{\sigma}_{ev}=\lambda^{\sigma}_{neo}$ and $\lambda^{\sigma}_{ev}=\lambda^{\sigma}_{pre}$, respectively, diffuse with a coefficient D_σ, are taken up by both normal cells (with a rate $\lambda^{\sigma}_{tissue}$) and tumor cells ($\lambda^{\sigma}_{tumor}$ in the proliferating region and q_s in the hypoxic region), and decay (with a rate λ^{σ}_{N}) in the necrotic region. Assuming steady-state conditions, the formulation is [4,7,8]:

$$0=\nabla\cdot\left(D_\sigma\nabla\sigma\right)+\lambda^{\sigma}_{ev}(\mathbf{x},t,\mathbf{1}_{vessel},p_i,\sigma,h)-\lambda^{\sigma}(\sigma)\sigma \qquad (4)$$

$$\lambda_p=\begin{cases}\lambda^{\sigma}_{tissue} & \text{outside}\,\Omega\\ \lambda^{\sigma}_{tumor} & \text{in }\Omega_P\\ q_\sigma(\sigma) & \text{in }\Omega_H\\ \lambda^{\sigma}_{N} & \text{in }\Omega_N\end{cases} \qquad (5)$$

where $\mathbf{x}$ is position in space, t is time, $\mathbf{1}_{vessel}$ is the characteristic function for vasculature (equals 1 at vessel locations and 0 otherwise), p_i is the interstitial pressure, and h is the hematocrit in the vascular network which is related to oxygen extravasation (following [4]). The extravasation is modulated by the extravascular interstitial pressure p_i scaled by the effective pressure p_e, with k_{p_i} being the weight of the convective transport component of small molecules [7]:

$$\lambda^{\sigma}_{ev}=\overline{\lambda}^{\sigma}_{ev}\mathbf{1}_{vessel}(\mathbf{x},t)\left(\frac{h}{\overline{H}_D}-\overline{h}_{min}\right)^{+}\left(1-k_{p_i}\frac{p_i}{p_e}\right)(1-\sigma) \qquad (6)$$

Constants $\overline{H}_D$ and h_{min} represent normal and minimum blood hematocrit required for extravasation, respectively, and $\overline{\lambda}_{ev}^{\sigma}$ is the constant vascular extravasation transfer rate from both pre-existing and tumor-induced vessels. The parameters in Equation 4 are calibrated so that the growth rate as an avascular spheroid matches the experimentally observed rate.

Extra-cellular matrix

The transport of diffusible substances within the stroma of tumor tissue is modulated by the ECM density E represented by [3]:

$$\frac{\partial E}{\partial t} = \overline{\lambda}_{production}^{E} \frac{1}{1+k_p E} \mathbf{1}_{\Omega_V} + \overline{\lambda}_{sprout.production}^{E} \frac{1}{1+k_p E} \mathbf{1}_{sprout.tips} - \overline{\lambda}_{degradation}^{E} \frac{EM}{1+k_d E} \quad (7)$$

where $\overline{\lambda}_{production}^{E}$ and $\overline{\lambda}_{sprout.production}^{E}$ are the rate of production by proliferating tumor tissue and sprouting capillary vessels during angiogenesis, respectively, and $\overline{\lambda}_{degradation}^{E}$ is the degradation rate. These rates are modulated by the existing ECM density. The degradation is further modulated by the density M of matrix degrading enzymes (MDE), which are released by proliferating tumor cells and vascular endothelial cells to remodel the ECM. The MDE concentration is [3]:

$$\frac{\partial M}{\partial t} = \nabla \cdot (D_M \nabla M) + \overline{\lambda}_{production}^{M} (1-M) \mathbf{1}_{\Omega_V} + \overline{\lambda}_{sprout.production}^{M} (1-M) \mathbf{1}_{sprout.tips} - \overline{\lambda}_{degradation}^{M} \frac{EM}{1+k_d E} - \overline{\lambda}_{decay}^{M} M \quad (8)$$

where D_T is the diffusion coefficient, $\overline{\lambda}_{production}^{M}$ and $\overline{\lambda}_{sprout.production}^{M}$ are the rate of production by viable tumor tissue and sprouting capillary vessels, respectively, $\overline{\lambda}_{degradation}^{T}$ is the rate of degradation as they are uptaken by the ECM, and $\overline{\lambda}_{decay}^{M}$ is the decay rate.

Transport of drug

The transport of drug s through tumor tissue, like oxygen, is also simulated from the location of extravasation from the vasculature. The drug diffuses through the tissue (water volume fraction) with a coefficient D_s. The uptake by tumor and normal cells and the wash-out from the interstitial space are included as a combined effect in the rate $\overline{\lambda}_{uptake}^{s}$, which reflects the drug half-life (assumed as a first approximation to be similar to the half-life in plasma):

$$0 = \nabla \cdot \left(D_s \nabla s \right) + \overline{\lambda}_{ev}^{s}(\mathbf{x}, t, \mathbf{1}_{vessel}, p_i, s) - \overline{\lambda}_{ECM.uptake}^{s} Es - \overline{\lambda}_{uptake}^{s} s \qquad (9)$$

Assuming a constant vascular extravasation transfer rate $\overline{\lambda}_{ev}^{s}$ from both pre-existing and tumor-induced vessels, the drug extravasation is:

$$\lambda_{ev}^{s} = \overline{\lambda}_{ev}^{s} \mathbf{1}_{vessel}(\mathbf{x}, t)(1 - k_{p_i} \frac{p_i}{p_e})(\frac{C_t^s}{\overline{C}^s} - s) \qquad (10)$$

where the diffusion in tumor tissue is assumed to be modulated by the interstitial pressure [7]. The drug concentration in the vasculature is initially $\overline{C}^{s}$, with the extravasation assumed to be of the form $C_t^s = \overline{C}^{s} e^{-\alpha t}$. This assumes first order kinetics, for which the extravasation is mainly concentration dependent. The decay α is based on an average half-life of 1 hour for Gem (for short infusion [9]).

The boundary condition for all the diffusion equations is $\frac{\partial B}{\partial n} = 0$ (zero Neumann condition), where B is the diffusible substance.

Drug effect on the tumor

The drug only affects proliferating cells in order to simulate the cell-cycle dependent effects of Gem. Accordingly, the drug effect is included into the proliferation term λ_p, where $\overline{\lambda}_{effect}$ is the rate of drug-induced cell death [8]:

$$\lambda_p = \begin{cases} 0 & \text{outside } \Omega \\ \lambda_{\mathrm{M}} \sigma (1 - \overline{\lambda}_{effect} s) - \lambda_{\mathrm{A}} & \text{in } \Omega_P \\ 0 & \text{in } \Omega_H \\ -G_N & \text{in } \Omega_N \end{cases} \qquad (11)$$

where λ_M is the mitosis rate, λ_A is the apoptosis rate, and G_N is the non-dimensional rate of volume loss in the necrotic regions assuming that cellular debris is constantly degraded and the resulting fluid is removed [8]. The model assumes that the cellular proliferation and apoptosis rates are comparable prior and after therapy. The cellular death process is assumed to last 1d after exposure to a cytotoxic concentration of drug. We first calibrated the model parameters for tumor growth, oxygen, and drug effect to obtain a simulated reduction in size for an avascular tumor spheroid matching *in vitro* data from [10] with PANC-1 cells. We then used this drug effect to simulate the therapy on a lesion *in vivo*.

Radio Frequency effect on the tumor

The effect of RF treatment is included in the model by scaling the drug diffusive penetration in the tissue according to the experimental measurements with Capan-1 tumor spheroids showing a 75% increase in molecule penetration when the spheroids are pre-treated with RF (see **Table 1**). It is also observed in the experiments that the tissue becomes more permeable post treatment; to simulate this effect, the blood vessel permeability (which affects the drug vascular extravasation transfer rate from the vasculature) is scaled with the same factor as the drug diffusive penetration, assuming as a first approximation that the tissue and blood vessel permeability scale similarly. To simulate the effect of resistance after multiple RF treatments, based on a decline in 10% in the cell population exhibiting roundness after four RF treatments [2] , the model implements at 2.5% decrease in the drug effect after each RF exposure.

Supplementary Table 1: Main parameters of the computational model and their associated values. All other parameters are as in [3].
(*) Value is rescaled by the square of the simulation system characteristic length (1 cm) and divided by the system characteristic time (1 sec) multiplied by the oxygen diffusive penetration [11] (1×10^{-5} cm^2 s^{-1}). Gem: gemcitabine.

**Parameter**	**Value**	**Reference**
Tumor proliferation rate	1 day^{-1}	Measured from experimental data (monolayer cell culture)
Tumor necrosis threshold	0.5500	Calibrated to simulate pancreatic lesion
Tumor hypoxic threshold	0.5750	Calibrated to simulate pancreatic lesion
Oxygen diffusive penetration	1 (*)	[3]
Oxygen transfer rate from vasculature	5 (*)	[3]
Oxygen uptake rate by proliferating tumor cells	1.5 (*)	[3]
Oxygen uptake rate by hypoxic tumor cells	1.3 (*)	[3]
Oxygen uptake rate by tumor microenvironment	0.12 (*)	[3]
Oxygen decay rate	0.35 (*)	[3]
Gem diffusive penetration	0.2 (*)	Measured from experimental data
Gem transfer rate from vasculature	5 (*)	[7]
Gem cellular uptake rate	1.5 (*)	[7]
Gem diffusive penetration after RF	0.35 (*)	Measured as 75% higher than without RF
Gem transfer rate from vasculature after RF	8.75 (*)	Scaled as Gem penetration after RF
Gem cellular uptake rate after RF	1.5 (*)	Assumed same as without RF
Gem drug effect	4.1×10^4	Calibrated to experimental data [10]
Gem half life	60 min.	42-94 min. for short infusion [9]
Viability differential between Gem and RF plus Gem for PANC-1 (monolayer)	1.00 : 0.74	Measured for 100 μM Gem for 24hr
Gem in vitro EC70 (72 hrs.) for PANC-1 (spheroid)	100 μM	[10]
Gem dose *in vivo* (human): 1000 mg/m^2	6.3×10^{-4} kg/L for typical adult	[9]

SUPPLEMENTARY FIGURES

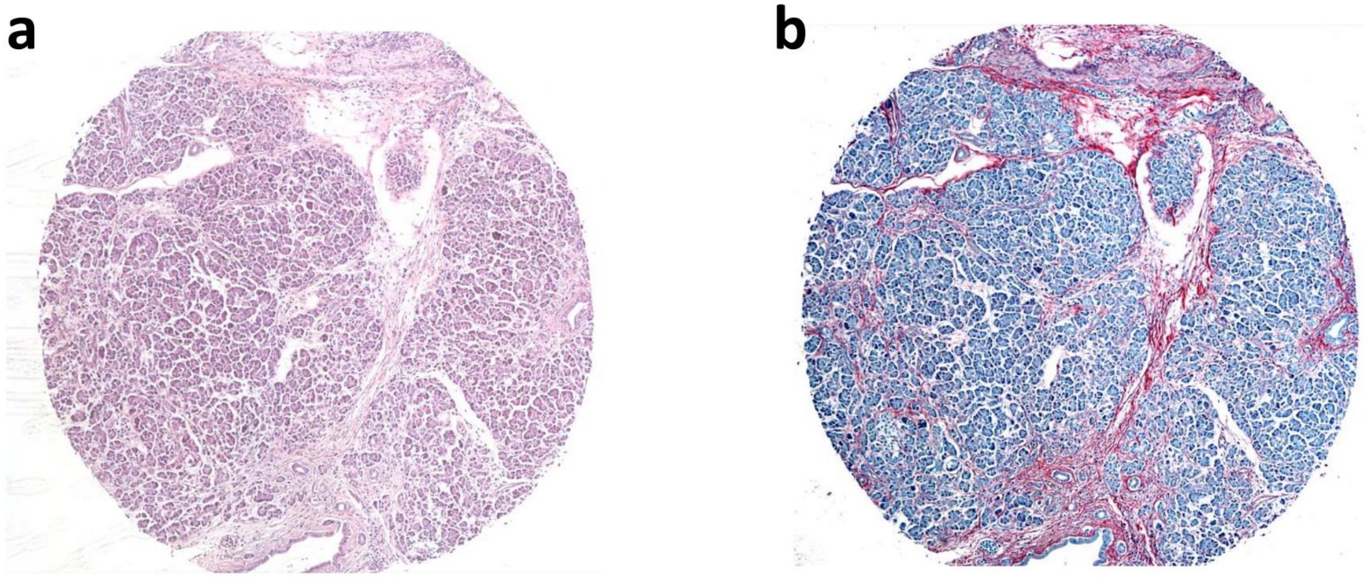

Supplementary Figure 1: Pancreatic tumor samples reveal non-uniform presence of stroma. Shown are human PDAC stained for (A) H&E and (B) collagen.

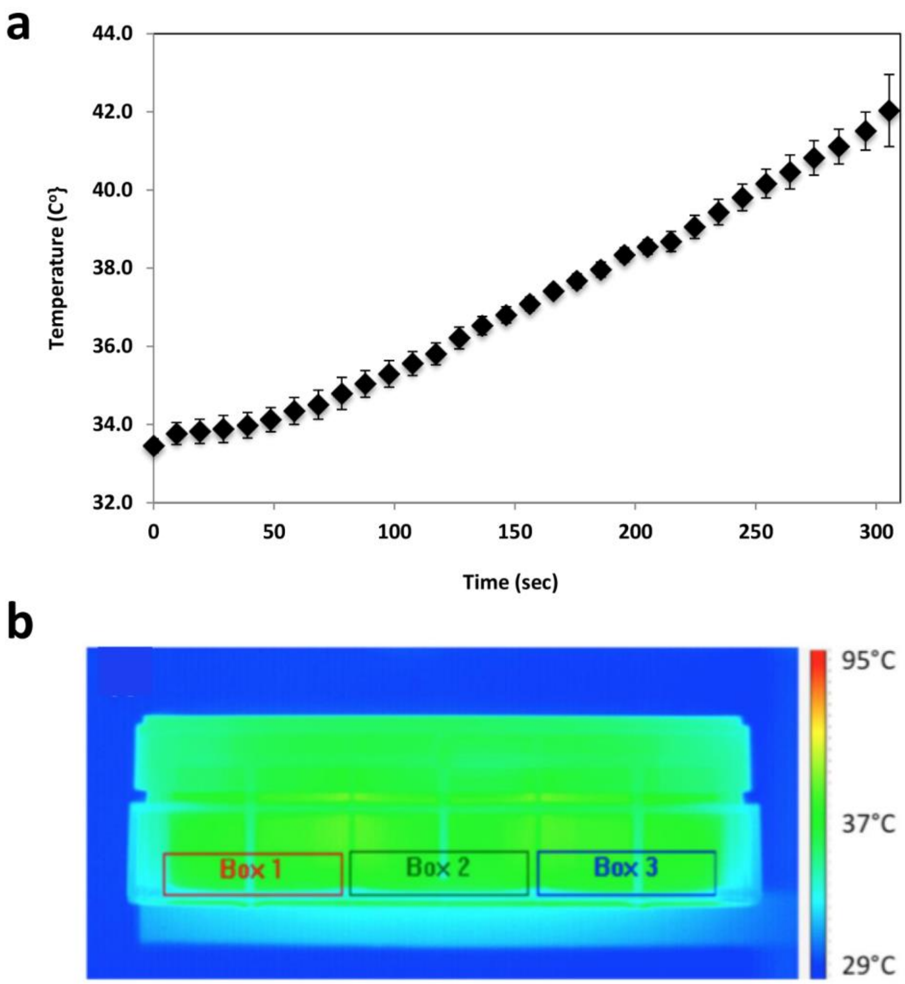

Supplementary Figure 2: Measurement of temperature in 3D cell culture samples during RF exposure using an infrared camera. (A) Slight rise in temperature over 300 sec. during RF exposure. (B) Infrared imaging of cell culture plate shows no discernable undue rise in temperature during exposure to RF.

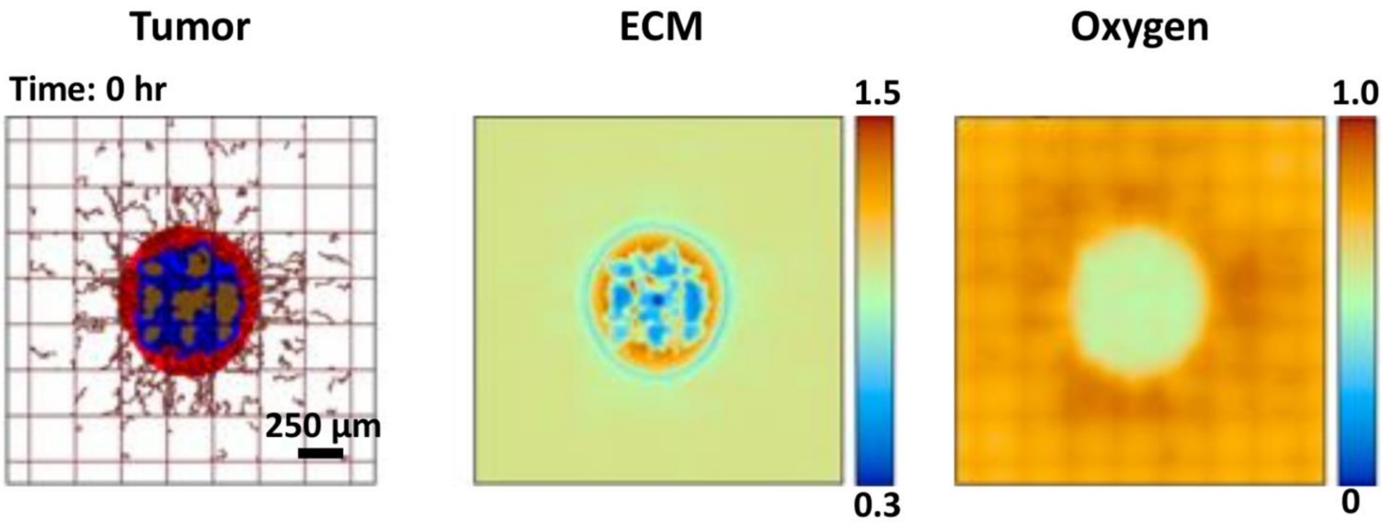

Supplementary Figure 3: Simulated pancreatic tumor lesion prior to treatment. (A) Tumor tissue includes proliferating (red), hypoxic (blue) and necrotic (brown) regions. Existing capillary network is denoted by regularly spaced grid (brown), with vessels induced by angiogenesis shown as irregular lines growing towards the hypoxic tumor regions which act as a source of angiogenic stimuli. Vascular flow is from bottom left to upper right of each panel. Normal tissue (not shown) surrounds the lesion. (B) Extra-cellular matrix (ECM) concentration (non-dimensional units) corresponding to the lesion in (A), and of sufficient density to affect the drug diffusive penetration. (C) Oxygen concentration profile due to diffusion gradients maintained by heterogeneous vascularization (with a maximum value normalized by the vascular concentration), highlighting the lower oxygen inside most of the lesion compared to the surrounding host tissue. Tumor diameter is ~800 µm.

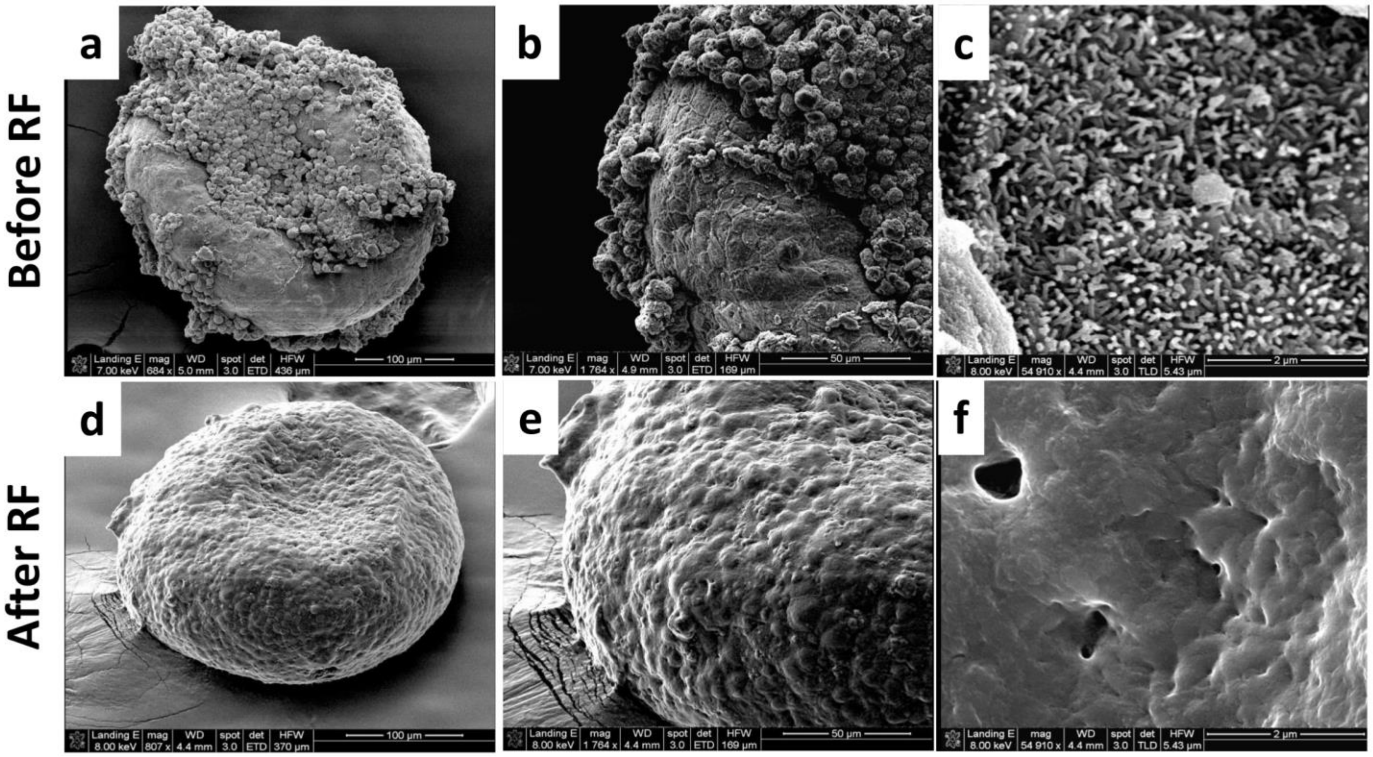

Supplementary Figure 4: Capan-1 spheroids before and 0 h after RF treatment. Top row: SEM images of a Capan-1 spheroid before RF treatment at increasing magnifications (left 684X, middle 1,764X and right 54,910X). Bottom row: SEM images of a Capan-1 spheroid 0h after RF treatment at increasing magnifications (left 807X, middle 1,764X and right 54,910X). The higher magnification middle and right panels highlight the “smoothening” of the spheroid surface and the formation of pores following RF treatment.

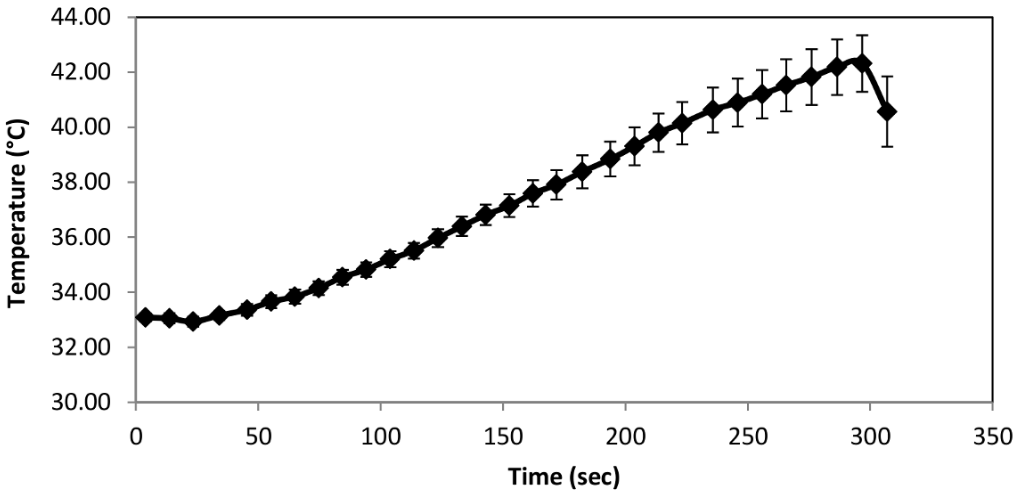

Supplementary Figure 5: Calibration of the RF field for the cell culture experiments. Data indicate mean ± SD, n=3.

REFERENCES

1 Ware, M. J. *et al.* Generation of homogeneous 3D pancreatic cancer cell spheroids using an improved hanging drop technique. *Tissue Eng. C* **in press**, (2016).

2 Ware, M. J. *et al.* Radiofrequency treatment alters cancer cell phenotype. *Sci. Rep.* **5**, 12083, (2015).

3 Wu, M. *et al.* The effect of interstitial pressure on tumor growth: Coupling with the blood and lymphatic vascular systems. *J. Theor. Biol.* **320**, 131-151, (2013).

4 Macklin, P. *et al.* Multiscale modelling and nonlinear simulation of vascular tumour growth. *J. Math. Biol.* **58**, 765-798, (2009).

5 McDougall, S. R., Anderson, A. R. A. & Chaplain, M. A. J. Mathematical modelling of dynamic adaptive tumour-induced angiogenesis: Clinical implications and therapeutic targeting strategies. *J. Theor. Biol.* **241**, 564-589, (2006).

6 Macklin, P. *et al.* Multiscale modelling and nonlinear simulation of vascular tumour growth. *J. Math. Biol.* **58**, 765-798, (2009).

7 van de Ven, A. L. *et al.* Integrated intravital microscopy and mathematical modeling to optimize nanotherapeutics delivery to tumors. *AIP Adv.* **2**, 11208, (2012).

8 Wu, M. *et al.* The effect of interstitial pressure on therapeutic agent transport: Coupling with the tumor blood and lymphatic vascular systems. *J. Theor. Biol.* **355**, 194-207, (2014).

9 Gemzar <http://www.drugs.com/pro/gemzar.html> (2017).

10 Wen, Z. *et al.* A spheroid-based 3-D culture model for pancreatic cancer drug testing, using the acid phosphatase assay. *Braz. J. Med. Biol. Res.* **46**, 634-642, (2013).

11 Nugent, L. J. & Jain, R. K. Extravascular diffusion in normal and neoplastic tissues. *Cancer Res.* **44**, 238-244, (1984).
